# Patterns of functional connectivity alterations induced by alcohol reflect somatostatin interneuron expression in the human cerebral cortex

**DOI:** 10.1038/s41598-022-12035-5

**Published:** 2022-05-12

**Authors:** Ryo Ochi, Fumihiko Ueno, Mutsuki Sakuma, Hideaki Tani, Sakiko Tsugawa, Ariel Graff-Guerrero, Hiroyuki Uchida, Masaru Mimura, Shunji Oshima, Sachio Matsushita, Shinichiro Nakajima

**Affiliations:** 1 Department of Neuropsychiatry, Keio University School of Medicine, 35 Shinanomachi, Shinjuku-ku, Tokyo, 160-8582 Japan; 2 Multimodal Imaging Group, Research Imaging Centre, Centre for Addiction and Mental Health, Toronto, ON Canada; 3 National Hospital Organization Kurihama Medical and Addiction Center, Kanagawa, Japan; 4 Sustainable Technology Laboratories, Asahi Quality and Innovations, Ltd., Ibaraki, Japan

**Keywords:** Neuronal physiology, Biomarkers

## Abstract

Acute alcohol administration affects functional connectivity, yet the underlying mechanism is unknown. Previous work suggested that a moderate dose of alcohol reduces the activity of gamma-aminobutyric acidergic (GABAergic) interneurons, thereby leading to a state of pyramidal disinhibition and hyperexcitability. The present study aims to relate alcohol-induced changes in functional connectivity to regional genetic markers of GABAergic interneurons. Healthy young adults (N = 15, 5 males) underwent resting state functional MRI scanning prior to alcohol administration, immediately and 90 min after alcohol administration. Functional connectivity density mapping was performed to quantify alcohol-induced changes in resting brain activity between conditions. Patterns of differences between conditions were related to regional genetic markers that express the primary GABAergic cortical interneuron subtypes (parvalbumin, somatostatin, and 5-hydroxytryptamine receptor 3A) obtained from the Allen Human Brain Atlas. Acute alcohol administration increased local functional connectivity density within the visual cortex, sensorimotor cortex, thalamus, striatum, and cerebellum. Patterns of alcohol-induced changes in local functional connectivity density inversely correlated with somatostatin cortical gene expression. These findings suggest that somatostatin-expressing interneurons modulate alcohol-induced changes in functional connectivity in healthy individuals.

## Introduction

Alcohol is one of the most widely used addictive substances in the world. A subset of individuals who drink alcohol develop alcohol use disorder (AUD), which is a chronically relapsing disorder and accounts for 5% of deaths globally^1^. The global consequences of AUD include 3 million annual deaths^2^. According to estimates from 2006, the global economic burden of alcohol is between $210 billion and $650 billion^3^. In light of this large societal impact, there is an urgent demand to identify how alcohol use alters brain function. Although its rewarding and anxiolytic effects are well-known, neurobiological mechanisms underlying alcohol-induced physiological changes still remain unclear. A deeper understanding of the underlying mechanisms is expected to identify pathways that play a fundamental role in the pathophysiology of developing AUD.

Much of the research of acute alcohol challenge examined the impact on brain function measured by functional magnetic resonance imaging (fMRI). Studies measuring resting-state functional connectivity reported that acute alcohol administration was related to increased functional connectivity within the visual cortex, striatum, and thalamus^4–7^. These findings are not thought to reflect ethanol-induced vasodilatory effects because the affected areas are mainly located within the frontal and temporal regions^8–10^. Furthermore, Shokri-Kojori et al. demonstrated that alcohol-induced increases in functional connectivity of healthy participants were related to changes in mood effects, disrupted motor function, and declines in cognitive performance^11–14^. Thus, measures derived from fMRI may closely reflect the regional sensitivity to alcohol administration. However, the underlying basis of inter-regional variations in the sensitivity to alcohol is still undiscovered.

Alcohol generally facilitates gamma-aminobutyric acid (GABA)-A receptor function and inhibits glutamate receptors, thereby disrupting the balance between excitatory and inhibitory neurotransmissions^15–17^. Specifically, a moderate dose of alcohol leads GABAergic interneurons to a hypoactive state by diminishing intrinsic excitability of GABAergic interneurons, which results in disinhibition of pyramidal neurons and hyperexcitability^18–20^. Supporting this, previous work demonstrated that alcohol inhibited the kainate receptor-dependent excitatory drive of GABAergic interneurons in the hippocampus^19^. Evidence has suggested that inhibition of pyramidal neurons, led by GABAergic interneurons is critical for curbing alcohol consumption behaviors^18,21,22^. GABAergic interneurons comprise three major distinct subtypes in the diverse morphology, connectivity, and physiology: parvalbumin (PVALB), somatostatin (SST), and 5-hydroxytryptamine 3a receptors (HTR3A)^23,24^. Although these cellular processes are thought to underlie alcohol-induced changes in functional connectivity^4,25,26^, there currently is little understanding of the translational link between cellular and neuroimaging signatures in humans.

The recent emergence of the field of imaging transcriptomics makes it possible to yield new insights into the relationship between inter-regional variations in gene expression and neuroimaging phenotypes of interest^27,28^. In this field, the Allen Human Brain Atlas (AHBA) is frequently used and contains more than 20,000 genes taken from 3702 brain areas in MRI-derived stereotactic space^29^. Previous studies using the AHBA examined the transcriptional correlates of pharmacologically-induced changes in functional connectivity^30–33^. However, no studies examined the relationships between alcohol-induced changes in functional connectivity and specific gene expression. Uncovering these relationships would enhance our understanding of the mechanism of action of alcohol and ultimately lead to the development of intervention strategies for AUD.

In this study, we aimed to characterize neurobiological mechanisms underlying alcohol-induced changes in functional connectivity. First, resting-state fMRI data from healthy participants undergoing alcohol administration were used to generate the profile of inter-regional variations in the sensitivity to alcohol. We adopted an alcohol clamp technique^34,35^, which can reduce experimental variances due to inter-individual differences in alcohol pharmacokinetics. Furthermore, to eliminate the possibility of reflecting the alcohol’s chronic effect, we only included young healthy adults. Next, to achieve the aim, we related alcohol-induced changes in functional connectivity to the gene expression data from the AHBA. Based on the alcohol’s effects on GABAergic interneurons^18–20^, cortical expression maps for PVALB, SST, and HTR3A were correlated with alcohol-induced changes in resting-state functional connectivity. We hypothesized that alcohol-induced changes in functional connectivity correlated with the expression of one or more GABAergic interneuron subtypes.

## Method

### Participants

This study was approved by the institutional review board of the National Hospital Organization Kurihama Medical and Addiction Center and Keio University School of Medicine. Participants provided written informed consent statements in accordance with the declaration of Helsinki before participation in the study.

Participants were recruited through online advertisements and underwent a screening visit before inclusion. Inclusion criteria were: aged between 20 and 30 years, right-handed, non-smoker, occasional drinker (i.e., up to twice a month), carrying aldehyde dehydrogenase (ALDH) 2*1/*2 allele of the ALDH2 gene, and no current or past major medical conditions confirmed with by a self-report and normal results in liver enzymes quantification (i.e., aspartate aminotransferase < 33 IU/L, alanine aminotransferase < 42 IU/L, and gamma-glutamyl transferase < 47 IU/L). Notably, to minimize the inter-individual variability of the sensitivity to alcohol, we only included those carrying ALDH2*1/*2. The exclusion criteria included: carrying the ALDH2*1/*1 genotype that is less sensitive to alcohol or ALDH2*2/*2 that would result in extreme sensitivity or intolerance to alcohol, conditions impeding an MRI examination, taking any medication, a history of any allergies, and a history of drug dependence or drug abuse confirmed with a self-report and urine drug screening.

Eighteen participants initially took part in the study. Three participants were excluded due to the excessive head motion (defined as mean framewise displacement (mFD) being greater than 0.3 mm) during at least one MRI scan. As a result, a sample of 15 participants was included in the final analysis (n = 5 males and n = 10 females; mean age = 25.1 years; standard deviation (SD) = 2.9 years).

### Experimental design

**Figure 1:**
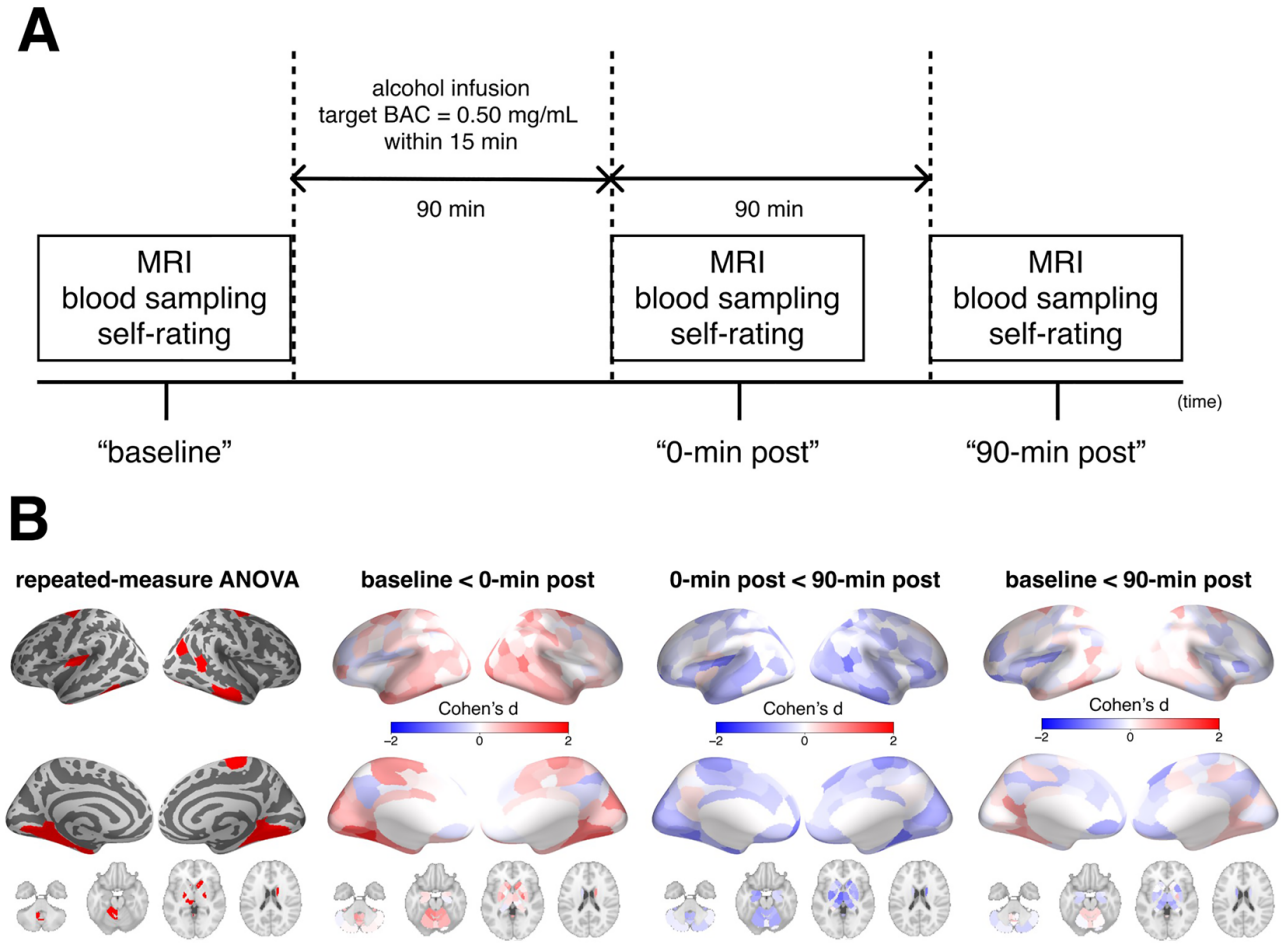
Experimental design and the effect on functional connectivity. Panel (**A**) represents the experimental design of the present study. Panel (**B**) represents differences of log-transformed local functional connectivity density across the conditions. The leftmost column of the panel shows regions which have significant differences across the conditions revealed by repeated-measure ANOVA. The others show regional effect sizes (Cohen’s d) of the differences of log-transformed local functional connectivity density between the conditions. These maps are visualized using pysurfer (0.10) (https://github.com/nipy/PySurfer). *ANOVA* analysis of variance, *BAC* blood alcohol concentration.

Each participant visited the National Hospital Organization Kurihama Medical and Addiction Center at two different days. At the first visit, participants completed a set of assessments of demographic characteristics and genetics. Besides ALDH2, we assessed ADH1B genotype, which is related to alcohol metabolism^36^. This study procedures on the second visit of each participant. Participants were instructed to refrain from taking alcohol for 24 h and eating for 6 h prior to the 8:30 am study start. The alcohol clamp technique was used to maintain the target blood alcohol concentration (BAC)^34,35^. To minimize experimental variance in BAC levels, 6% (volume/volume) alcohol in Ringer’s lactate solution was administered intravenously according to a PBPK model. The infusion profile of alcohol solution was precomputed using a PBPK model of an individual’s alcohol distribution and elimination to follow the desired time course of BAC, i.e., linear ascension to a target BAC of 0.50 mg/mL at 15 min, followed by a steady BAC maintained within 0.05 mg/mL of the target until a steady-state basis for calculating the alcohol elimination rate was established^37^. These parameters were based on previous literature that adopted the same methodology and examined the acute effects of intravenous alcohol on cognitive function^38^. Breath alcohol concentration (BrAC) was obtained to estimate BAC every minute until a plateau in BAC was reached and then every 5 min after that throughout the experiment. At the end of alcohol clamping, the average BrAC of participants was 0.245 mg/L (standard deviation was 0.014), analogous to 0.49 mg/mL of BAC. BrAC was measured using an Alco-Sensor^®^ IV (Intoximeter, Inc., St. Louis, MO, USA). A set of assessments including MRI scans and self-report mood/drug effects was performed prior to alcohol administration (the “baseline” condition) and immediately and 90 min after alcohol administration (the “0-min post” and “90-min post” conditions, respectively) (Fig. 1A). We did not ask participants to perform any tasks during MRI acquisition. Self-report alcohol effects were scored using visual analogue scale from 0 (not at all) to 10 (extremely) for feelings of 14 domains. These feelings were classified into 2 categories (stimulant or sedation) based on the Biphasic Alcohol Effects Scale^39^ (for details, please see Supplementary Table 1). Average values within each category were calculated as a summary score and used for the analysis.

### Neuroimaging data acquisition

MRI data were acquired on a 3 T MRI scanner (MR750; GE Discovery, Milwaukee, WI, USA) equipped with a 12-channel head coil at Kurihama Medical and Addiction Center. Resting-state fMRI data were acquired using an echo-planar imaging sequence (echo time = 30 ms, repetition time = 2500 ms, flip angle = 80°, field of view = 212 × 212 mm^2^, matrix size = 64 × 64, voxel size = 3.3 × 3.3 × 4.0 mm, slice thickness = 3.2 mm, 240 volumes, 40 axial slices). During acquisition of resting-state fMRI data, participants were instructed to lie quietly with their eyes open and fixated on a crosshair. Also, for image processing, T1-weighted whole brain anatomical data were acquired using a BRAVO sequence (echo time = 3.064 ms, repetition time = 7.028 ms, inversion time = 650 ms, flip angle = 8°, field of view = 256 × 256 mm^2^, matrix size = 256 × 256, slice thickness = 0.9 mm, 200 sagittal slices).

### Image processing

Preprocessing of T1-weighted anatomical and resting-state fMRI data underwent a standard volumetric preprocessing pipeline using fMRIPrep 20.2.1^40^, which is based on Nipype 1.5.1^41^. A full description of the preprocessing pipeline can be found in the Supplementary material. Briefly, the pipeline included the following steps: slice-timing-correction, motion correction, skull stripping, and nuisance estimation. Finally, preprocessed data were then resampled via nonlinear transformation to the MNI152NLin2009cAsym standard volumetric space.

Following fMRIPrep, the blood-oxygen-level-dependent (BOLD) time series in the standard space were further denoised to eliminate the impact of in-scanner head motions and non-neuronal confounds on BOLD time series. We employed a recommended strategy^39^, which involved regressing out (1) 2 physiological time series (mean signal in cerebrospinal fluid and that in white matter), (2) global signal, (3) 6 motion parameters representing 3 translation and 3 rotation time series, (4) temporal derivatives of (1–3), (5) quadratic terms of (1–4), and (6) frames exceeding a motion threshold (0.5 mm FD) as a spike regressor. This strategy was shown to be relatively effective for reducing motion-related artifacts^42–44^. Notably, we chose to include global signal as one of confounds because global signal regression was proposed to attenuate non-neuronal artifacts (i.e., breathing rate and vigilance), which persist across sessions of each participant in pharmacological fMRI studies^30,45,46^. Finally, denoised BOLD data were band-pass filtered (0.01–0.1 Hz) and spatially smoothed with a 6 mm full-width at half maximum Gaussian kernel.

To characterize alcohol-induced changes in brain function, we estimated local functional connectivity density (lFCD), quantifying the extent of spatial synchrony in BOLD signal fluctuations to index spontaneous brain activity demand^47^. Tomasi and Volkow have demonstrated that the variability of lFCD is low (12%), suggesting that this metric is a reliable measure of brain functional connectivity^47^. Pearson’s correlation was calculated to assess the strength of functional connectivity between two voxels. As prior studies^4,47,48^, a positive correlation threshold of r = 0.6 was used to compute the binary correlation coefficients to ensure that significant correlations between time-varying signal fluctuations are connected. Then, lFCD of each voxel was calculated as the size of a continuous cluster of voxels above the threshold that are connected by surface. Because degree-related measures follow an exponential distribution, we used log(lFCD) with a semi-normal distribution. Finally, log(lFCD) data were parcellated into 200 cortical regions of the Schaefer 7-network based volumetric atlas^49^, 32 subcortical regions^50^, and 28 cerebellar regions^51^. Since mFD was significantly different across the conditions (p = 0.015), we examined relationships between in-scanner motion as indexed by mFD and log(lFCD). The mean correlation coefficient of the relationships was 0.138, implying less motion-related effects on log(lFCD).

### Gene expression maps

The AHBA is a publicly available resource containing whole-brain microarray gene expression data obtained from post-mortem tissue samples of six adult human donors^29^. Human gene expression microarray data were extracted from the AHBA using the abagen toolbox (version 0.1.3; https://github.com/netneurolab/abagen^52^ with default parameters. Gene expression data were parcellated into 200 cortical regions of the Schaefer volumetric atlas^49^. To archive the aim of present study, we specifically generated gene expression maps of GABAergic interneuron subtypes (PVALB, SST, and HTR3A).

### Statistical analysis

For behavioral measures, alcohol-induced changes of stimulant and sedation scores across conditions were compared using repeated-measure analysis of variance (ANOVA). To characterize alcohol-induced changes in functional connectivity, regional log(lFCD) values were compared using repeated-measure ANOVA. Results were corrected for multiple comparisons using the false discovery rate Benjamini–Hochberg method. For significant regions, post-hoc comparisons were performed using Bonferroni correction. Differences of log(lFCD) of the significant regions across conditions were compared between groups divided based on sex and ADH1B genotypes. Furthermore, as an exploratory analysis, differences of log(lFCD) of the significant regions across conditions were related to differences of alcohol’ stimulant and sedation scores across conditions. As a main analysis, the inter-regional profiles of alcohol-induced physiological changes were related to inter-regional expression profiles of each of GABAergic interneuron subtypes (PVALB, SST, and HTR3A) by estimating Pearson’s correlation coefficients. The present study focused only on the cortex due to well-documented differences in the transcriptional signatures of the cortex, subcortex, and cerebellum^53^. To assess the significance of the relationship, we used spatial autocorrelation-preserving permutation tests that generated random surrogate brain surface maps. Using BrainSMASH toolbox, we shuffled gene expression maps while maintaining spatial autocorrelation to generate 5000 surrogate brain maps for each of gene expression profiles^54^. P values were estimated from the null distribution of Pearson’s correlation coefficients calculated from the surrogate map and the effect size (Cohen’s d) of log(lFCD) differences and were corrected for multiple comparisons using the false discovery rate Benjamini–Hochberg method.

## Results

### Alcohol alters whole-brain local functional connectivity density and induces subjective effects

The main effect of alcohol on log(lFCD) revealed significant differences across conditions in cortical, subcortical, and cerebellar regions (Fig. 1B). These regions included the visual, sensorimotor, and temporal cortices as well as the thalamus, striatum, and cerebellum. The area most affected was the left visual association cortex (Vis_1; F(2,28) = 21.663, P_FDR_ < 0.001). Post-hoc comparisons revealed that log(lFCD) of these regions was increased at the 0-min post condition relative to the baseline condition and was reduced at the 90-min post condition relative to the 0-min post condition (for details of region names and statistical information, please see Supplementary Table 2). Differences of log(lFCD) across the conditions were not different in terms of sex and ADH1B genotypes (all p’s > 0.1).

For behavioral measures, the sedative score was different across the conditions (p < 0.001) while the stimulant score was not (p > 0.9). Specifically, the sedation score was increased in both 0-min post and 90-min post conditions relative to the baseline condition (0-min post; p < 0.001, 90-min post; p = 0.02, respectively). Furthermore, within the regions significantly affected by alcohol, the degree of increased log(lFCD) of the only one region (the left temporal pole within the limbic network) correlated with the decreased sedative score in the 0-min post condition relative to the baseline condition (r =  − 0.52, p = 0.0049).

### Alcohol-induced changes in functional connectivity correlate with SST cortical gene expression

**Figure 2:**
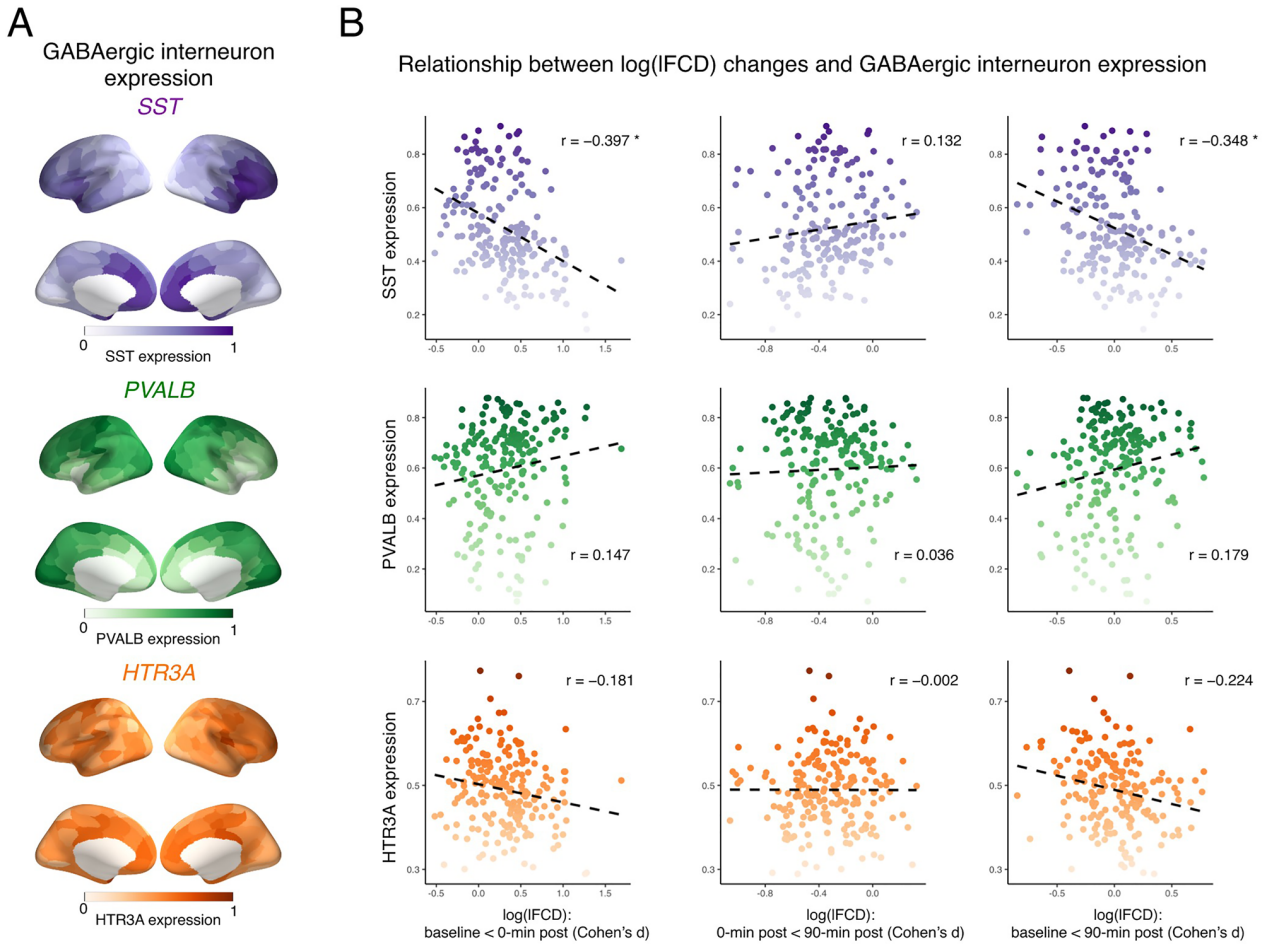
Correlation between alcohol-induced changes in functional connectivity and cortical expression maps specific to GABAergic interneuron subtypes. Panel (**A**) shows distributions of cortical expression of each GABAergic interneuron subtype. These cortical expression data are visualized using pysurfer (0.10) (https://github.com/nipy/PySurfer). Panel (**B**) shows spatial relationships between log(lFCD) changes across conditions and cortical gene expression specific to each GABAergic interneuron subtype. *P_FDR_ < 0.05. *HTR3A* 5-hydroxytryptamine 3a receptor, *lFCD* local functional connectivity density, *GABA* gamma-aminobutyric acid, *PVALB* parvalbumin, *SST* somatostatin.

To further investigate the mechanism of action of alcohol, we tested whether alcohol-induced log(lFCD) changes were related to inter-regional gene expression profiles specific to GABAergic interneuron subtypes (Fig. 2). We found that patterns of log(lFCD) differences between baseline and 0-min post conditions inversely correlated with SST cortical gene expression (r =  − 0.397, p = 0.029). This is also the case for degrees of log(lFCD) differences between baseline and 90-min post conditions (r =  − 0.348, p = 0.025). There were no relationships between alcohol-induced log(lFCD) changes and cortical gene expression of other GABAergic interneuron subtypes.

## Discussion

**Figure 3:**
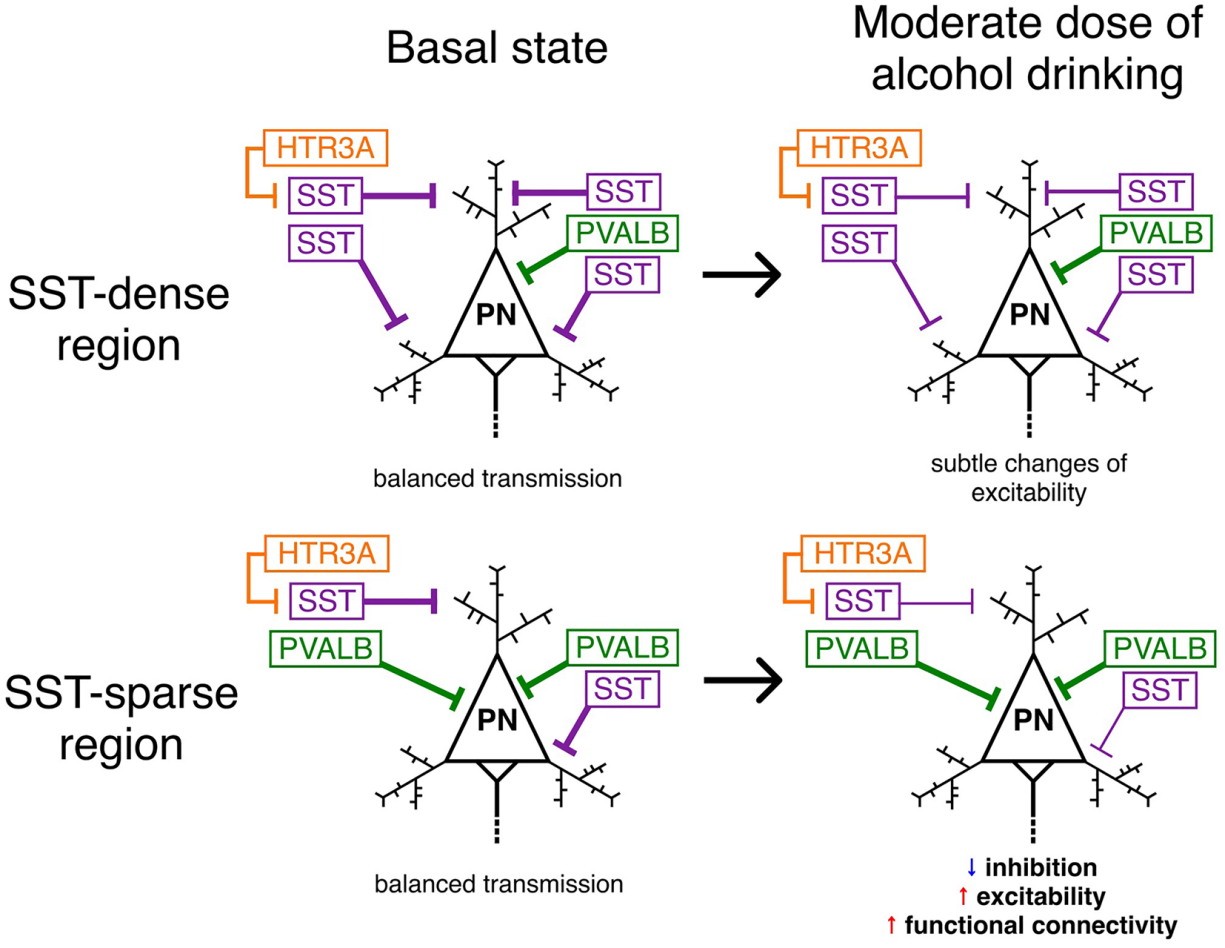
A proposed model of alcohol-induced changes in functional connectivity. In basal states, pyramidal neurons are inhibited by GABAergic interneurons, which leads to balanced transmissions. After drinking a moderate dose of alcohol, the activity of SST-expressing interneurons is disrupted, resulting in disinhibition, hyperexcitability, and increased functional connectivity only in SST-sparse regions. *HTR3A* 5-hydroxytryptamine 3a receptor, *GABA* gamma-aminobutyric acid, *PN* pyramidal neuron, *PVALB* parvalbumin, *SST* somatostatin.

Here we characterized the neurobiological basis underlying acute alcohol-induced changes in functional connectivity. Given that these changes would vary with individuals’ characteristics, we recruited participants so that all participants in this study shared similar characteristics of age, genetics, and drinking habits. The present study enhances our understanding of the mechanism of action of alcohol by showing that (1) acute alcohol increases log(lFCD) across regions including visual cortex, temporal cortex, thalamus, striatum, and cerebellum; (2) the increased log(lFCD) is normalized in the 90-min post condition; (3) log(lFCD) in the left temporal pole is related to sedative effects of alcohol; (4); patterns of log(lFCD) changes after acute alcohol administration inversely correlate with SST cortical gene expression. Overall, our findings suggest the role of SST-expressing interneurons as a key modulator of alcohol-induced changes of local functional connectivity (Fig. 3).

We found that acute alcohol administration induced hyper-connectivity within the unimodal cortices (visual cortex and sensorimotor cortex) and subcortical regions (thalamus and striatum), and cerebellum, suggesting increased neuronal activities following alcohol administration. Previous studies measuring cerebral blood flow during alcohol intoxication found that alcohol increased the metric particularly within the frontal and temporal cortices^8–10^, suggesting that our findings may reflect changes in neuronal activities rather than neurovascular effects. In line with our findings, Shokri-Kojori et al. found that acute alcohol administration increased log(lFCD) within the thalamus^4^. Previous studies reported that alcohol increased intrinsic connectivity within the visual network^5,6^. In addition, we found that alcohol-induced log(lFCD) changes in the left temporal pole inversely correlated with changes in the sedation score, suggesting that increased log(lFCD) in the unimodal cortex may lead to alcohol-induced behavioral effects. Since the temporal pole belongs to the limbic network^55,56^, individuals with increased log(lFCD) in this region after alcohol administration may have greater stimulated effects than sedative effects. Given the region is sensitive to signal loss caused by susceptibility artifacts, future studies that replicate this relationship are warranted. Notably, network hierarchy from unimodal to transmodal cortices is a key principle of brain organization^57,58^, and is disrupted in various disorders related to sensory processing^59,60^. Thus, acute alcohol administration may disrupt network hierarchy by increasing neuronal activity in the unimodal cortex, resulting in suboptimal information flow and behavioral changes. Collectively, acute alcohol administration increases local functional connectivity in the unimodal cortex, subcortex, and cerebellum, which may induce behavioral effects of alcohol.

Most importantly, patterns of acute alcohol-induced changes in log(lFCD) relative to the baseline condition are inversely correlated with SST cortical gene expression. In other words, regions with high degrees of alcohol-induced log(lFCD) increases relative to the baseline condition had a low density of SST-expressing interneurons. This finding supports the central role of SST-expressing interneurons in alcohol’s neuronal and subjective effects^18^. In general, SST-expressing interneurons regulate the activity of excitatory pyramidal neurons by targeting their dendrites^23,61^. These interneurons are synaptically connected to nearby pyramidal neuron and densely wired into local neuronal networks^61^. Recent in vivo work noted that a moderate dose of alcohol reduced the activity of SST-expressing interneurons, which results in disinhibition of pyramidal neurons^20^. Additionally, chronic ethanol exposure leads to increased intrinsic excitability of pyramidal neurons in the prefrontal cortex (PFC) in mices^62,63^. The directionality of our findings may reflect that SST-sparse regions, such as the unimodal cortices, are sensitive to a moderate dose of alcohol whereas SST-dense regions, such as the PFC, are not. Thus, excessive alcohol use may disrupt the activity of SST-expressing interneurons in the PFC and lead to pyramidal disinhibition (Fig. 3), and ultimately may increase risk of compulsive alcohol drinking and alcohol dependence in humans^18^. That is, drinking enough to affect SST-expressing interneurons in the PFC may shift individuals from controlled to compulsive alcohol seeking. Supporting this, evidence demonstrated that excitatory glutamatergic inputs from the PFC to the striatum contributed to compulsive alcohol seeking and taking^64^. Dysfunction of the fronto-striatal circuits is of interest in various mental illnesses that are related to impaired inhibitory control^65–67^. Taken together, alcohol-induced changes in log(lFCD) may reflect the activity of SST-expressing interneurons, which can be used as personalized biomarkers of transitioning from moderate to excessive alcohol use. Future studies with a larger sample size and high-risk for AUD participants are warranted to identify the relationships between alcohol-induced changes in functional connectivity and known risk factors for developing AUD^68,69^.

The present study should be considered in light of several limitations. First, the sample size was relatively small. Second, the intravenous alcohol injection seems not to be same experience with alcohol drinking. Third, although we carefully removed confounds of the BOLD signal, mFD was significantly changed across the conditions. Fourth, the present study is based on small samples of postmortem brains and more comprehensive microarray gene expression atlases are warranted for future studies. Finally, the mean age and ethnicity of participants, those of which would affect gene expression^70,71^, in the present study (mean age = 25.1 years; race = Asian) were not matched with that of donors of the AHBA (mean age = 43 years; race = Caucasian).

Using functional connectivity density mapping and whole-brain microarray gene expression data, we examined the effect of acute alcohol administration on the functional connectivity and its relationships with expression of GABAergic interneuron subtypes. A moderate dose of alcohol increased local functional connectivity in the unimodal cortices, subcortical regions, and cerebellum. To our knowledge, the present study provides first evidence that patterns of alcohol-induced changes in functional connectivity inversely correlate with SST cortical gene expression maps in humans. These results suggest the central role of SST-expressing interneurons in alcohol’s neuronal and subjective effects. Furthermore, alcohol-induced log(lFCD) changes may be used as personalized biomarkers of transitioning from moderate to excessive alcohol use. Future studies relating alcohol-induced changes in functional connectivity and participants’ characteristics may aid in furthering our understanding of risk factors for AUD and problematic alcohol use.

## Supplementary Information

Supplementary Information.
