# Supplementary material for: Patterns of functional connectivity alterations induced by alcohol reflect somatostatin interneuron expression in the human cerebral cortex
Source: Sci Rep. 2022 May 12;12:7896. doi: 10.1038/s41598-022-12035-5 (PMC9098480; doi:10.1038/s41598-022-12035-5)
Supplement: Supplementary file 1 — Supplementary Information. [file 41598_2022_12035_MOESM1_ESM.docx]

### Supplemental Materials

**fMRI preprocessing**

Functional images were pre-processed using fMRIPrep 20.2.1 [1] which is based on Nipype 1.5.1 [2].

Each T1-weighted volume was corrected for intensity non-uniformity and skull-stripped [3,4]. Brain tissue segmentation of cerebrospinal fluid (CSF), white-matter (WM) and gray-matter (GM) was performed on the brain-extracted T1w using fast (FSL 5.0.9) [5]. A T1w-reference map of each participant was computed after registration of 3 T1w images across conditions (after intensity non-uniformity-correction) using mri_robust_template (FreeSurfer 6.0.1) [6], and the brain mask estimated previously was refined with a custom variation of the method to reconcile ANTs-derived and FreeSurfer-derived segmentations of the cortical gray-matter of Mindboggle [7]. Volume-based spatial normalization to ICBM 152 Nonlinear Asymmetrical template version 2009c (MNI152NLin2009cAsym) [8] was performed through nonlinear registration with antsRegistration (ANTs 2.3.3), using brain-extracted versions of both T1w reference and the T1w template.

For each of the 3 BOLD runs found per subject (across all conditions), the following preprocessing was performed. First, a reference volume and its skull-stripped version were generated using a custom methodology of fMRIPrep. Susceptibility distortion correction (SDC) was omitted. The BOLD reference was then co-registered to the T1w reference using bbregister (FreeSurfer) which implements boundary-based registration [9]. Co-registration was configured with six degrees of freedom. Head-motion parameters with respect to the BOLD reference (transformation matrices, and six corresponding rotation and translation parameters) are estimated before any spatiotemporal filtering using mcflirt (FSL 5.0.9) [10]. BOLD runs were slice-time corrected using 3dTshift from AFNI 20160207 [11]. The BOLD time-series (including slice-timing correction when applied) were resampled onto their original, native space by applying the transforms to correct for head-motion. These resampled BOLD time-series will be referred to as preprocessed BOLD in original space, or just preprocessed BOLD. The BOLD time-series were resampled into standard space, generating a preprocessed BOLD run in MNI152NLin2009cAsym space. First, a reference volume and its skull-stripped version were generated using a custom methodology of fMRIPrep. Several confounding time-series were calculated based on the preprocessed BOLD: framewise displacement (FD), DVARS and three region-wise global signals. FD was computed using two formulations following Power (absolute sum of relative motions) [12] and Jenkinson (relative root mean square displacement between affines) [10]. FD and DVARS are calculated for each functional run, both using their implementations in Nipype (following the definitions by Power and colleagues [12]). The three global signals are extracted within the CSF, the WM, and the whole-brain masks. The head-motion estimates calculated in the correction step were also placed within the corresponding confounds file. The time series derived from head motion estimates and global signals were expanded with the inclusion of temporal derivatives and quadratic terms for each [13]. Frames that exceeded a threshold of 0.5 mm FD were annotated as motion outliers. All resamplings can be performed with a single interpolation step by composing all the pertinent transformations (i.e. head-motion transform matrices, susceptibility distortion correction when available, and co-registrations to anatomical and output spaces). Gridded (volumetric) resamplings were performed using antsApplyTransforms (ANTs), configured with Lanczos interpolation to minimize the smoothing effects of other kernels [14]. Non-gridded (surface) resamplings were performed using mri_vol2surf (FreeSurfer).

**Supplemental Table 1 – Domains and categories of self-report questionnaire**

|  | domain | category |
| --- | --- | --- |
| 1 | up | stimulant |
| 2 | stimulated | stimulant |
| 3 | energized | stimulant |
| 4 | excited | stimulant |
| 5 | talkative | stimulant |
| 6 | pleasant | stimulant |
| 7 | vigorous | stimulant |
| 8 | difficulty concentrating | sedative |
| 9 | slow thoughts | sedative |
| 10 | down | sedative |
| 11 | heavy head | sedative |
| 12 | inactive | sedative |
| 13 | sedated | sedative |
| 14 | tired | sedative |

**Supplemental Table 2 – Significant regions associated with alcohol administration and details of statistical analyses.**

| region | hemisphere | network | Mean (baseline) | Mean (0-min post) | Mean (90-min post) |
| --- | --- | --- | --- | --- | --- |
| Vis_1 | left | Visual | -0.089 | 0.382 | 0.106 |
| Vis_4 | left | Visual | -0.126 | 0.449 | -0.002 |
| Vis_6 | left | Visual | -1.141 | -0.604 | -0.811 |
| SomMot_3 | left | Somatomotor | -0.261 | -0.197 | -0.381 |
| SomMot_14 | left | Somatomotor | -1.000 | -0.728 | -0.886 |
| Post_1 | left | Dorsal Attention | -0.041 | 0.158 | 0.109 |
| TempPole_1 | left | Limbic | 0.084 | 0.238 | 0.041 |
| PHC_1 | left | Default | -0.302 | -0.004 | -0.152 |
| Vis_2 | right | Visual | 0.028 | 0.372 | 0.136 |
| Vis_3 | right | Visual | 0.374 | 0.662 | 0.475 |
| Vis_4 | right | Visual | -0.173 | 0.106 | -0.032 |
| Vis_7 | right | Visual | -0.995 | -0.690 | -0.839 |
| SomMot_16 | right | Somatomotor | -0.686 | -0.476 | -0.576 |
| SomMot_18 | right | Somatomotor | 0.024 | 0.296 | -0.069 |
| TempOccPar_1 | right | Ventral Attention | 0.163 | 0.397 | 0.174 |
| Par_1 | right | Default | 0.465 | 0.747 | 0.549 |
| Temp_2 | right | Default | 0.613 | 0.792 | 0.588 |
| Nac-core | right | subcortex | 1.414 | 1.794 | 1.395 |
| pGP | right | subcortex | 0.597 | 0.711 | 0.649 |
| aCAU | right | subcortex | 1.440 | 1.708 | 1.469 |
| pCAU | right | subcortex | 1.747 | 2.003 | 1.642 |
| THA.VP | left | subcortex | 1.231 | 1.278 | 1.082 |
| THA.DA | left | subcortex | 1.751 | 1.913 | 1.544 |
| NAc-shell | left | subcortex | 0.656 | 0.932 | 0.607 |
| pGP | left | subcortex | 1.186 | 1.302 | 1.129 |
| I / IV | left | cerebellum | -0.017 | 0.195 | 0.041 |
| V | left | cerebellum | 0.810 | 1.168 | 0.892 |
| VIIIb | left | cerebellum | 1.688 | 2.027 | 1.807 |
| Vermis_VIIIb | both | cerebellum | 1.611 | 1.887 | 1.636 |
| VIIIb | right | cerebellum | 1.666 | 1.964 | 1.725 |

| region | F | pFDR | Cohen's d (baseline < 0-min post) | Cohen's d (0-min post < 90-min post) | Cohen's d (baseline < 90-min post) |
| --- | --- | --- | --- | --- | --- |
| Vis_1 | 21.663 | 0.001 | 1.689 | -0.985 | 0.705 |
| Vis_4 | 10.202 | 0.025 | 1.279 | -0.747 | 0.252 |
| Vis_6 | 6.437 | 0.043 | 0.810 | -0.475 | 0.524 |
| SomMot_3 | 8.694 | 0.026 | 0.356 | -1.000 | -0.766 |
| SomMot_14 | 8.087 | 0.027 | 1.031 | -0.481 | 0.659 |
| Post_1 | 8.584 | 0.026 | 1.024 | -0.253 | 0.779 |
| TempPole_1 | 7.257 | 0.035 | 0.792 | -0.910 | -0.194 |
| PHC_1 | 7.851 | 0.027 | 0.996 | -0.492 | 0.549 |
| Vis_2 | 11.308 | 0.025 | 1.038 | -1.063 | 0.365 |
| Vis_3 | 7.704 | 0.027 | 1.261 | -0.559 | 0.346 |
| Vis_4 | 6.593 | 0.043 | 0.999 | -0.484 | 0.433 |
| Vis_7 | 7.608 | 0.027 | 1.023 | -0.540 | 0.471 |
| SomMot_16 | 8.560 | 0.026 | 1.018 | -0.466 | 0.663 |
| SomMot_18 | 7.749 | 0.027 | 0.799 | -0.802 | -0.301 |
| TempOccPar_1 | 10.329 | 0.025 | 1.012 | -1.042 | 0.047 |
| Par_1 | 7.192 | 0.035 | 0.864 | -0.643 | 0.340 |
| Temp_2 | 6.881 | 0.042 | 0.635 | -0.785 | -0.207 |
| Nac-core | 8.713 | 0.026 | 0.840 | -0.955 | -0.051 |
| pGP | 7.365 | 0.035 | 1.026 | -0.495 | 0.479 |
| aCAU | 6.419 | 0.043 | 0.987 | -0.591 | 0.112 |
| pCAU | 10.184 | 0.025 | 0.879 | -1.042 | -0.331 |
| THA.VP | 6.708 | 0.042 | 0.199 | -0.956 | -0.713 |
| THA.DA | 7.855 | 0.027 | 0.386 | -1.299 | -0.563 |
| NAc-shell | 7.569 | 0.027 | 0.715 | -0.977 | -0.152 |
| pGP | 8.209 | 0.027 | 0.746 | -0.966 | -0.333 |
| I / IV | 8.023 | 0.027 | 0.910 | -0.655 | 0.364 |
| V | 9.686 | 0.026 | 0.998 | -0.728 | 0.351 |
| VIIIb | 6.365 | 0.043 | 0.746 | -0.564 | 0.487 |
| Vermis_VIIIb | 6.654 | 0.042 | 0.743 | -0.696 | 0.115 |
| VIIIb | 6.513 | 0.043 | 0.798 | -0.650 | 0.224 |

**References**

1. Esteban, Oscar, Christopher J. Markiewicz, Ross W. Blair, Craig A. Moodie, A. Ilkay Isik, Asier Erramuzpe, James D. Kent, et al. 2019. “fMRIPrep: A Robust Preprocessing Pipeline for Functional MRI.” Nature Methods 16 (1): 111–16.

2. Gorgolewski, Krzysztof, Christopher D. Burns, Cindee Madison, Dav Clark, Yaroslav O. Halchenko, Michael L. Waskom, and Satrajit S. Ghosh. 2011. “Nipype: A Flexible, Lightweight and Extensible Neuroimaging Data Processing Framework in Python.” Frontiers in Neuroinformatics 5 (August): 13.

3. Tustison, Nicholas J., Brian B. Avants, Philip A. Cook, Yuanjie Zheng, Alexander Egan, Paul A. Yushkevich, and James C. Gee. 2010. “N4ITK: Improved N3 Bias Correction.” IEEE Transactions on Medical Imaging 29 (6): 1310–20.

4. Avants, B. B., C. L. Epstein, M. Grossman, and J. C. Gee. 2008. “Symmetric Diffeomorphic Image Registration with Cross-Correlation: Evaluating Automated Labeling of Elderly and Neurodegenerative Brain.” Medical Image Analysis 12 (1): 26–41.

5. Zhang, Y., M. Brady, and S. Smith. 2001. “Segmentation of Brain MR Images through a Hidden Markov Random Field Model and the Expectation-Maximization Algorithm.” IEEE Transactions on Medical Imaging 20 (1): 45–57.

6. Reuter, Martin, H. Diana Rosas, and Bruce Fischl. 2010. “Highly Accurate Inverse Consistent Registration: A Robust Approach.” NeuroImage 53 (4): 1181–96.

7. Klein, Arno, Satrajit S. Ghosh, Forrest S. Bao, Joachim Giard, Yrjö Häme, Eliezer Stavsky, Noah Lee, et al. 2017. “Mindboggling Morphometry of Human Brains.” PLoS Computational Biology 13 (2): e1005350.

8. Fonov, V. S., A. C. Evans, R. C. McKinstry, C. R. Almli, and D. L. Collins. 2009. “Unbiased Nonlinear Average Age-Appropriate Brain Templates from Birth to Adulthood.” NeuroImage Supplement 1 (47): S102.

9. Greve, Douglas N., and Bruce Fischl. 2009. “Accurate and Robust Brain Image Alignment Using Boundary-Based Registration.” NeuroImage 48 (1): 63–72.

10. Jenkinson, Mark, Peter Bannister, Michael Brady, and Stephen Smith. 2002. “Improved Optimization for the Robust and Accurate Linear Registration and Motion Correction of Brain Images.” NeuroImage 17 (2): 825–41.

11. Cox, R. W., and J. S. Hyde. 1997. “Software Tools for Analysis and Visualization of fMRI Data.” NMR in Biomedicine 10 (4-5): 171–78.

12. Power, Jonathan D., Anish Mitra, Timothy O. Laumann, Abraham Z. Snyder, Bradley L. Schlaggar, and Steven E. Petersen. 2014. “Methods to Detect, Characterize, and Remove Motion Artifact in Resting State fMRI.” NeuroImage 84 (January): 320–41.

13. Satterthwaite, Theodore D., Mark A. Elliott, Raphael T. Gerraty, Kosha Ruparel, James Loughead, Monica E. Calkins, Simon B. Eickhoff, et al. 2013. “An Improved Framework for Confound Regression and Filtering for Control of Motion Artifact in the Preprocessing of Resting-State Functional Connectivity Data.” NeuroImage 64 (January): 240–56.

14. Lanczos, C. 1964. “Evaluation of Noisy Data.” Journal of the Society for Industrial and Applied Mathematics Series B Numerical Analysis 1 (1): 76–85.
